# Early detection of peripheral neuropathy using stimulated skin wrinkling test in human immunodeficiency virus infected patients: A cross-sectional study

**DOI:** 10.1097/MD.0000000000011526

**Published:** 2018-07-27

**Authors:** Arthur H.P. Mawuntu, Corry N. Mahama, Herlyani Khosama, Riwanti Estiasari, Darma Imran

**Affiliations:** a Neurology Department Faculty of Medicine Sam Ratulangi University/R.D. Kandou Hospital, North Sulawesi; b Neurology Department, Faculty of Medicine, Universitas Indonesia/Cipto Mangunkusumo Hospital, Jakarta, Indonesia.

**Keywords:** brief peripheral neuropathy screening, eutectic mixture of local anesthetic, HIV peripheral neuropathy, Indonesia., stimulated skin wrinkling test

## Abstract

Peripheral neuropathy is a common condition of human immunodeficiency virus (HIV)-infected patients, which often remains undetected. We assessed the performance of stimulated skin wrinkling-eutectic mixture of local anesthetic (SSW-EMLA) test compared with brief peripheral neuropathy screening (BPNS) to detect HIV neuropathy.

This is a cross-sectional study conducted in HIV-positive patients. A modified skin wrinkling grading was used to assess SSW-EMLA effect. BPNS-detectable neuropathy was assessed by a combination of neuropathy severity scoring scale (subjective) and objective method of sensory and tendon reflex examination. The SSW-EMLA test accuracy with reference to BPNS was assessed using sensitivity and specificity and predictive values.

In a total of 99 HIV patients, 61.6% were males and the majority age group were between 30 and 40 years (52%). The neuropathy detection was SSW-EMLA test 36.4% versus BPNS 15.2% (*P* = .04). The sensitivity of SSW-EMLA test was 60.0% [95% confidence interval (95% CI) 34.5–81.7], specificity 67% (95% CI 63.3–3–71.7), and overall accuracy of 66.7% (95% CI 58.9–73.2).

The SSW-EMLA test detected many more peripheral neuropathy cases than BPNS in HIV patients and has potential as an alternative test for screening for HIV neuropathy in resource-constraint hospitals in Indonesia.

## 1 Introduction

Peripheral neuropathy (PN) is a common complication occurring in HIV-infected patients. The estimated global prevalence of human immunodeficiency virus (HIV) infection was approximately 38.8 million in 2015.^[1]^ The estimated PN prevalence among this group is 19% to 42% worldwide^[2–4]^ and it increases with age.^[2,4,5]^ The HIV-associated PN (HIV-PN) predominantly affects sensory nerve fiber symmetrically. The main pathological features associated with HIV-PN are distal axonal degeneration, loss of neurons in the affected dorsal root ganglia, infiltration of inflammatory cells, and a decrease in epidermal nerve fiber density. The clinical symptoms vary from severe pain, hypoesthesia, and weakness to asymptomatic.^[2]^ These conditions contribute to impairment of patient's daily activity function, which significantly decreases the quality of life.

The social and economic burden of HIV-PN is huge.^[1]^ Therefore, early detection is important for prompt management to prevent complications. There are 2 main techniques used to detect PN: invasive (skin biopsy) and noninvasive (clinical screening/examination methods, nerve ultrasound, and electroneurographic examination).^[6,7]^ Skin biopsy is an invasive method used to assess small nerve fiber condition by quantifying the epidermal nerve fiber density. This invasive method is considered a valuable diagnostic tool in various types of PN. However, this method is limited by special skill, tissue processing method, and optic equipment required to make a reliable result.^[7]^ Therefore, clinicians prefer to opt for noninvasive techniques for early detection of HIV-PN.^[6]^

Some of the commonly used noninvasive techniques are skin vasoconstrictor reflex (SVCR), neuromuscular ultrasound, Sudoscan, nerve conduction study (NCS), and brief peripheral neuropathy screening (BPNS). In SVCR method, PN is detected by determining the reduction in blood flow using a Doppler device over the digit tip skin during an inspiratory gasp. The limitation of this technique is it requires a strict observation of a controlled standardized environment, inspiratory gasp practice and technique by the patients, and the availability of laser Doppler, which is often unavailable in many centers.^[8]^ Neuromuscular ultrasound is a noninvasive technique that uses a high-resolution ultrasonographic device to obtain the image of the nerves.^[9,10]^ However, the weakness lies in its low-sensitivity, as past studies reported variations in the correlation between ultrasound findings and clinical findings.^[9,10]^ Sudoscan, a simple and quick method based on electrochemical reaction between sweat chloride and stainless-steel electrodes or electrochemical skin conductance for detecting small fiber neuropathy, can also be applied to various neurology cases. Its reliability and reproducibility have been investigated in some cases such as diabetes and leprosy. However, this method is not widely available in Indonesia and still requires a specific software.^[11–13]^ NCS is a well-known technique among clinical neurologist that uses an electroneurographic device to detect PN. Nonetheless, the sensitivity of NCS to detect small nerve fiber pathology is limited, while most early HIV-PN involves small nerve fibers.^[2]^ Furthermore, this method requires trained personnel and specialized equipment, which is not commonly available at provincial and district level hospitals. In these facilities, the frequently used method is BPNS. It is an affordable, fast, and easy test to screen HIV-PN, which is suitable for limited-resource settings. It uses a scoring system in assessing subjective and objective features of neuropathy to determine the likelihood of having PN in HIV patients.^[14]^ Yet, it is limited by low sensitivity (around 49%) to detect PN.^[15]^

The other test method, stimulated skin wrinkling (SSW) to detect PN in HIV patient, which is yet not widely used, is the focus of this study. SSW refers to the reversible undulations of the surface skin occurring 5 to 30 minutes following water immersion or exposure to a eutectic mixture of local anesthetic (EMLA). Skin wrinkling occurs because of vasoconstriction in the glabrous skin, mediated by post-ganglionic sympathetic fibers.^[16–18]^ EMLA affects the vasoconstriction by a passive diffusion to the digital pulp vasculature via the sweat glands.^[19]^ More recently, the use of SSW with EMLA (SSW-EMLA) has been reported in conditions with small fiber neuropathy.^[20]^ A study using SSW-EMLA for the detection of diabetic neuropathy found comparable sensitivity to other methods including NCS. Following receiver operating characteristic-analysis, at a cut-off point of <3 for an abnormal SSW-EMLA, sensitivity of SSW-EMLA for diagnosing diabetic neuropathy using NCS as a reference standard was 81.3%, and specificity was 67.0%.^[21]^

As diabetic and HIV-PN has similar pathology, we hypothesized that this test can also be applied to HIV patients. Furthermore, the low cost and easy to use SSW-EMLA method might be useful for early detection of HIV-PN in resource-limited hospitals if it is shown to have a better performance for detecting early neuropathy. We explored SSW-EMLA test performance for the detection of HIV-PN compared with the current method of BPNS in HIV patients.

## 2 Methods

The study was conducted in the HIV/AIDS outpatient clinics at the Cipto Mangunkusumo Hospital, Jakarta between June 2014 and February 2015. HIV was confirmed by HIV-positive enzyme-linked immunosorbent assay (ELISA) test documented in patient's medical records. The study's ethical clearance was given by the Ethical Committee of the Cipto Mangunkusumo hospital No. 407/H2.F1/ETIK/2014.

### 2.1 Study participants

HIV-positive patients, aged between 18 and 60 years who provided written informed consent, were enrolled in the study. Among these patients, we set the exclusion criteria and conducted its screening through interviews and reviewed of the medical record. We excluded patients with a history of systematic illness that could cause neuropathy, history of repetitive activities using hands, congenital anomalies of the hands, fractures or other injuries to arm or hands, peripheral vascular diseases or responses such as Raynaud phenomenon, an open wound in the area where EMLA would be applied, and clinical symptoms of myelopathy or radiculopathy. We also excluded patients with other potential conditions that could cause PN such as anemia, diabetes, thyroid disease, renal disease/uremia, liver disease, and malignancies. Patients who had received chemotherapeutic drugs, statins, amiodarone, and/or tacrolimus for more than 7 days were also excluded from the study due to the same reason. Once eligibility was confirmed and the patients consented, they underwent physical, anthropometric [height, weight, and body mass index measurement (BMI)], and neurological examinations (visual acuity, cranial nerves functions, motor strength and tone, reflexes, and sensory functions). Their medical records were checked to obtain results of HIV status, complete blood count, CD4 count, and viral load. Antiretroviral drugs (ARV) history was obtained from the medication list found in the HIV/AIDS clinic database and treatment adherence was confirmed through interviews.

### 2.2 Test procedures

We conducted BPNS and SSW-EMLA test sequentially, first the BPNS followed by SSW-EMLA test. There was a 30-minute interval between these 2 tests.

#### 2.2.1 BPNS

The BPNS protocol was adapted from AIDS Clinical Trial Group (ACTG) protocol.^[14]^ There are 2 examination components for conducting this technique: subjective and objective. First, patients were examined for the subjective components. It included checking for any complaints of pain, aches, or burning sensation in their feet or legs, needle or nail piercing sensation in their feet or legs, or numbness sensation in their feet or legs. If the patient had no complaints, we asked if they ever experienced those symptoms before the outpatient visit when the test was conducted. If they answered yes, we asked them to rate the severity of each symptom from mild to severe, ranging from a scale of 01 to 10. If the patient never experienced any of those symptoms, we scored 11 (always normal). If the symptoms had been present in the past, but not since the last visit, we scored 00 (currently absent). Each hand was scored separately. The highest severity score was used to count the subjective sensory neuropathy scale. The subjective sensory neuropathy scale was as follows: scale 01 to 03 scored as grade 1; scale 04 to 06 was noted as grade 2; scale 07 to 10 noted as grade 3; and scale 11 and 00 scored as grade 0.

The subjective component was followed by 2 objective components: vibratory sensation test on the distal interphalangeal (DIP) joints of the big toe using 128 Hz tuning fork; and deep tendon reflexes of the Achilles tendons test on both ankles using a reflex hammer.

For the vibratory sensation test, we vibrated a 128 Hz tuning fork and compressed the ends of the tuning fork just hard enough that the sides touched. The vibrating tuning fork was then placed on the bony prominence of the patient's wrist or hand to be sure that he/she could recognize the vibration or “buzzing” quality of the tuning fork. Again, we compressed the ends of the tuning fork just hard enough that the sides touched and immediately place the vibrating tuning fork gently, but firmly on the top of the DIP joint of one big toe and counted the seconds. We instructed the patient to tell us when the “buzzing” stopped. The time was recorded using a digital clock. The score was as follows: score 0 means the patient felt the vibration for >10 seconds (normal); score 1 was given if the patient felt the vibration for 6 to 10 seconds (mild loss); score 2 if the patient felt the vibration for ≤5 seconds (moderate loss); score 3 was given when the patient did not feel the vibration at all (severe loss); score 8 was given when the test is not done. We repeated the test on the other foot.

The deep tendon reflexes were tested on the Achilles tendons on each ankle. With the patient seated, we used 1 hand to press upward on the ball of the foot, dorsiflexed the patient's ankle to 90°. Using a reflex hammer, we then struck the Achilles tendon. The tendon reflex was felt by our hand as a plantar flexion of the foot, appearing after a slight delay from the time the Achilles tendon was hit. We used reinforcement by having the patient clench his/her fist before classifying the reflex as absent. The ACTG score was as follows: 0 – no reflex; 1 – hypoactive; 2 – normal; 3 – hyperactive; 4 – with clonus; and 8 when the test was not conducted.

The score for each BPNS test was summed up and interpreted as positive or negative for PN using the criteria described above. The BPNS result was considered positive for neuropathy if we found positive subjective component with at least 1 positive objective components.

#### 2.2.2 SSW-EMLA test

We used a modified method of SSW-EMLA test described by Ng et al.^[21]^ First, both hands of the patient were washed using soap and water and dried. The examiner observed the initial appearance of the fingers and photographed them before EMLA 5% cream (lidocaine 2.5% and prilocaine 2.5%; AstraZeneca; Cambridge, United Kingdom) application. A thick covering of EMLA 5% cream was applied to the distal segment of the 2nd, 3rd, and 4th fingers of both right and left hands. Each finger was wrapped in a food grade plastic wrap and sealed using skin bandage. After the application of EMLA cream, the patients were asked to wait for 30 minutes and to keep the hands dry and clean. The patients were not allowed to drink coffee, tea, or smoke cigarettes during the waiting period. If the EMLA application was compromised, the patient waited for an extra hour and the whole procedure was repeated.

**Figure 1:**
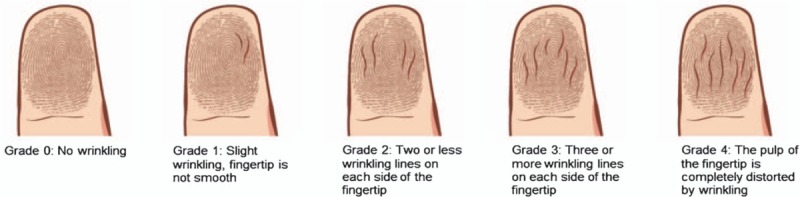
Skin wrinkling scale.

When 30 minutes were up, the examiner opened the bandages and examined the skin for wrinkling. The finger-tip appearances were compared with a special scale (Fig. 1) and scored. Wrinkling grades for digits 2, 3, and 4 were counted and average obtained. A difference of ≥3 points per hand (i.e., ≥1 point per digit) was taken as a cut-off or a different score. The SSW-EMLA test was considered abnormal if the total wrinkling score of each hand was <9.

### 2.3 Statistical analysis

Data were entered and analyzed using SPSS version 22 statistical software package (SPSS Inc., Chicago, IL). Categorical variables were analyzed and expressed as percentages. The SSW-EMLA test was compared with BPNS for neuropathy detection using sensitivity, specificity, positive, and negative predictive values. Univariate analysis was done using these following independent variables: gender, age, education, BMI, duration of ARV, adherence to ARV, lowest CD4+ count, history of stavudine therapy, history of zidovudine therapy, on stavudine therapy, and on zidovudine therapy with neuropathy as the dependent variable. To assess the significance of the differences between groups, we used the Chi-square test or Fisher exact test (for nonparametric variables). A *P* value of .05 was considered the threshold for statistical significance.

## 3 Results

In total, 99 HIV confirmed patients participated in the study. Nearly two-thirds of them were male; one-third were <30 years (33%), with the majority in the age of group between 30 and 40 years; and 84% had BMI > 18.6. Education level with greater than 9 years of schooling was high (97.0%). The majority of participants adhered to ARV treatment (87.9%) and 80.8% had a usage of ARV between 3 and 56 weeks. We also found 57.6% participants, CD4+ count was less than 100 cells/mm^3^, and 42.4% 101 and 500 cells/mm^3^.

### 3.1 Detection of neuropathy with SSW-EMLA test and BPNS

**Figure 2:**
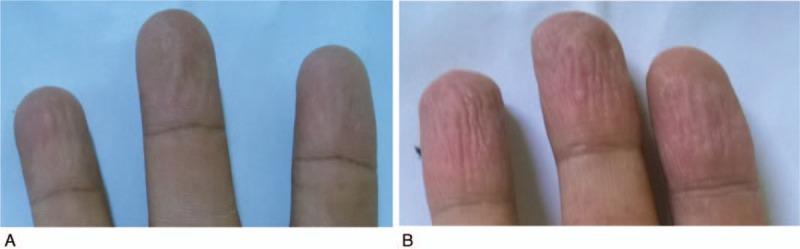
Sample of post-EMLA results. (A) Score 2 + 1 + 1 = 4, average 1.3 indicating a positive SSW-EMLA result; (B) Score 4 + 4 + 4 = 12, average 4, indicating a negative SSW-EMLA result.

The PN was defined by abnormal results of SSW-EMLA test (Fig. 2A) and BPNS was 36.4% (36/99) and 15.2% (15/99) (*P* = .04), respectively. The accuracy of SSW-EMLA test to detect PN compared with BPNS was 66.7% (the ability to differentiate PN taking into account the true positive and true negatives) with a sensitivity of 60.0% and 67.9% specificity (Table 1). There were 27 abnormal cases by SSW-EMLA test that were undetected by BPNS, with a 9.5% false negativity of SSW-EMLA test.

**Table 1:**
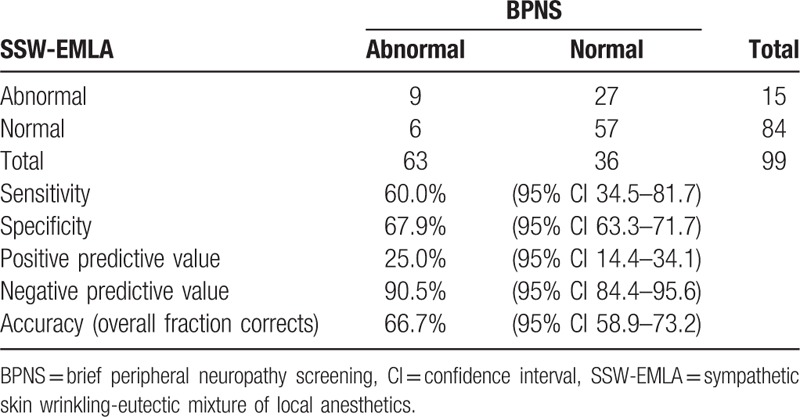
Correlation between SSW-EMLA and BPNS with diagnostic accuracy indices.

### 3.2 Variables associated with SSW-EMLA test and BPNS

Among the different variables studied, education was marginally associated with an abnormal SSW-EMLA test compared with patients with normal SSW-EMLA test (Table 2). Gender, age group, BMI, ARV duration, adherence to ARV, lowest CD4+ count, history of stavudine therapy, history of zidovudine therapy, currently on stavudine, and currently on zidovudine were not associated with abnormal SSW-EMLA test (*P* > .05). When analyzed for BPNS, age group >40 years has an association with abnormal BPNS (*P* = .02). There was no association with other variables and abnormal BPNS.

**Table 2:**
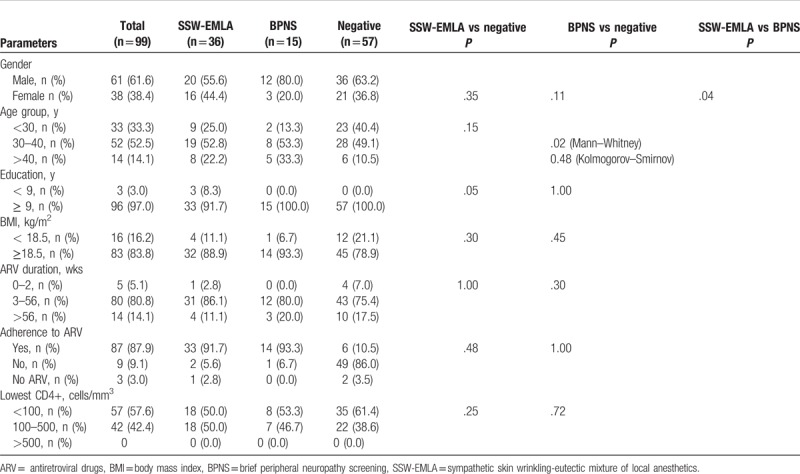
Variables associated with SSW-EMLA and BPNS.

## 4 Discussion

The performance of SSW-EMLA test was evaluated as a potential alternative to BPNS screening method to detect PN in HIV patients. We found SSW-EMLA test detected nearly 2 cases of PN for every case detected by BPNS, 36.4% and 15%, respectively. SSW-EMLA test picked up 27 cases that were considered normal by BPNS. We analyzed this finding correlated with the more objective characteristic of SSW-EMLA test and the nature of SSW that involve the small nerve fiber.

The correlation between small fiber neuropathy and SSW has been studied by some investigators and reviewed by Wilder-Smith.^[19]^ Compared with other commonly used indicator of small fiber neuropathy, the sympathetic skin response (SSR), SSW showed far superior results. Furthermore, when SSW was compared with Neuropads (a simple sweat test) in picking up small fiber neuropathy, SSW showed better sensitivity but lower specificity.^[19]^ The biological plausibility with EMLA is that it examines the autonomic nervous system signal transmission mediated by small nerve fibers. In chronic peripheral neuropathies (length-dependent), small nerve fibers are affected earlier than the larger fibers.^[19]^ In HIV patient, damage to the small fiber occurs as a result of the intake of nonreverse transcriptase inhibitors (NRTIs) drugs and neurotoxic effect of HIV as a result of glycoprotein 120 involvement.^[22]^

In our study, there was no association between gender and PN and is similar to previous studies.^[23,24]^ Likewise, we did not find an association with SSW-EMLA test in detecting PN in our study, although other studies have shown the incidence of HIV PN was associated with increased age.^[4]^ This is likely related to the poor immunological status found in our patients as shown in lower CD4+ finding. Decreased CD4+ count correlates with worsening immunological status, which is associated with neuropathy in HIV patients. All patients with abnormal SSW-EMLA test in our study had a CD4+ count of fewer than 500 cells/mm^3^ and 50% of them had CD4+ <100 cells/mm^3^. We also found no association between CD4+ count and PN. This can be explained by the nature of the research method (cross-sectional study) where the data were collected only at one point in time.

Our study is one of the first to use SSW-EMLA test to detect PN in HIV patients, and therefore, we could not compare it with other studies in HIV patients using the same method. However, 2 previous studies showed a higher sensitivity of SSW-EMLA test in detecting PN. First, a study on diabetic patients showed comparable sensitivity to other methods for detecting PN. Diabetic neuropathy and HIV PN have similarities, as both are affected by length-dependent neuropathy with early disruption of the axon and tendency to affect the small fibers first.^[21]^ Second, another study in Indonesia compared SSW-EMLA test and Sudoscan to detect autonomic neuropathy in multibacillary leprosy patients.^[25]^ The results revealed that SSW-EMLA test had higher sensitivity in comparison to Sudoscan, 64.3% and 32.9%, respectively. Like these studies, our study in HIV patients indicated a higher sensitivity of SSW-EMLA test in detecting PN in comparison to BPNS.

Our interest was to find a practical screening test that could be used in resource-constraint health facilities. Such a test should have an acceptable balance of easy usage, low false negative, and high accuracy. The SSW-EMLA test demonstrated these qualities comparable to our reference test, BPNS. The high NPV suggested that SSW-EMLA test correctly identified a patient who did not have a neuropathy.

The cross-sectional design limited us to assess associations of variables that change over time. In addition, we were unable to use a more sensitive test such as NCS. However, several studies have already validated SSW-EMLA test and shown it to be a reliable method for screening PN in conditions similar to HIV neuropathy.^[18]^ Our study was not designed to validate the SSW-EMLA test technique and therefore was limited to the use of 1 observer.

Compared with the BPNS, the SSW-EMLA test detected more cases and the detection of PN by the SSW-EMLA test was comparable to previous reports of HIV neuropathy by other methods. These findings indicated that SSW-EMLA test is a more objective and practical method than BPNS. Therefore, the method has potential as an alternative test for screening for HIV neuropathy in resource-constraint setting. Additional prospective studies are needed with more sensitive comparison tests to confirm the benefit of SSW-EMLA test for HIV neuropathy detection.

## Acknowledgment

We thank Rukhsana Ahmed and Ralalicia Limato for reviewing the statistical analysis made in this research.

## Author contributions

**Conceptualization:** Arthur H.P. Mawuntu, Darma Imran.

**Data curation:** Arthur H.P. Mawuntu, Corry N. Mahama.

**Formal analysis:** Arthur H.P. Mawuntu, Herlyani Khosama.

**Investigation:** Arthur H.P. Mawuntu, Herlyani Khosama.

**Methodology:** Arthur H.P. Mawuntu, Riwanti Estiasari.

**Project administration:** Corry N. Mahama.

**Resources:** Riwanti Estiasari.

**Software:** Arthur H.P. Mawuntu.

**Supervision:** Riwanti Estiasari, Darma Imran.

**Validation:** Riwanti Estiasari, Darma Imran.

**Visualization:** Arthur H.P. Mawuntu.

**Writing – original draft:** Arthur H.P. Mawuntu.

**Writing – review & editing:** Corry N. Mahama, Herlyani Khosama, Riwanti Estiasari, Darma Imran.
